# Glutenin and Gliadin, a Piece in the Puzzle of their Structural Properties in the Cell Described through Monte Carlo Simulations

**DOI:** 10.3390/biom10081095

**Published:** 2020-07-23

**Authors:** Joel Markgren, Mikael Hedenqvist, Faiza Rasheed, Marie Skepö, Eva Johansson

**Affiliations:** 1 Department of Plant Breeding, Swedish University of Agricultural Sciences, P.O. Box 101, SE-230 53 Alnarp, Sweden; joel.markgren@slu.se; 2 Department of Fibre and Polymer Technology, School of Engineering Sciences in Chemistry, Biotechnology and Health, KTH Royal Institute of Technology, SE-100 44 Stockholm, Sweden; mikaelhe@kth.se (M.H.); faizar@kth.se (F.R.); 3 Theoretical Chemistry, Lund University, P.O. Box 124, SE-221 00 Lund, Sweden; marie.skepo@teokem.lu.se

**Keywords:** modeling, intrinsically disordered proteins, gluten, disulfide bonds, cysteine, prolamin, Monte Carlo

## Abstract

Gluten protein crosslinking is a predetermined process where specific intra- and intermolecular disulfide bonds differ depending on the protein and cysteine motif. In this article, all-atom Monte Carlo simulations were used to understand the formation of disulfide bonds in gliadins and low molecular weight glutenin subunits (LMW-GS). The two intrinsically disordered proteins appeared to contain mostly turns and loops and showed “self-avoiding walk” behavior in water. Cysteine residues involved in intramolecular disulfide bonds were located next to hydrophobic peptide sections in the primary sequence. Hydrophobicity of neighboring peptide sections, synthesis chronology, and amino acid chain flexibility were identified as important factors in securing the specificity of intramolecular disulfide bonds formed directly after synthesis. The two LMW-GS cysteine residues that form intermolecular disulfide bonds were positioned next to peptide sections of lower hydrophobicity, and these cysteine residues are more exposed to the cytosolic conditions, which influence the crosslinking behavior. In addition, coarse-grained Monte Carlo simulations revealed that the protein folding is independent of ionic strength. The potential molecular behavior associated with disulfide bonds, as reported here, increases the biological understanding of seed storage protein function and provides opportunities to tailor their functional properties for different applications.

## 1. Introduction

The gluten protein complex is highly abundant in the wheat seed [1] and consists of storage proteins, which are known to form the largest protein networks in nature [2]. Similar types of proteins are found in other cereals and grasses (Poaceae) and are all genetically closely related and show high resemblance [3,4]. These proteins often share a high proline and glutamine content, amino acids lined in repetitive motifs, and cysteines (CYS) that form disulfide bonds that, in several cases, crosslink with other storage proteins [5]. Gluten proteins are divided into two groups: gliadins and glutenins [5,6]. Based on the amino acid composition, the gliadins are then further subdivided into three major classes, α-/β-, γ-, and ω-gliadins, whereas the glutenins are divided into low molecular weight (LMW) and high molecular weight (HMW) glutenin subunits (GS). Among these proteins, the gliadins and the LMW-GS have a similar evolutionary background; they share several features in their primary structure. Both types of protein contain regions of repetitive sequences, mainly of hepta- and dodecapeptides repeat motifs rich in proline and glutamine residues [7]. Even though the gliadins and the LMW-GS share similarities in their amino acid composition, their behavior of forming polymers differs in their native state, i.e., in the wheat grain. Gliadins are known as monomeric in their native state, with only intramolecular disulfide bonds (except for ω-gliadins due to the lack of CYS), while LMW-GS are known to form polymers by intermolecular disulfide bonds with other glutenin proteins, forming large polymers [8].

In the cell, both gliadins and glutenins are synthesized in the ribosomes of the rough endoplasmic reticulum (ER) and transported to ER lumen [9]. After this, the proteins form disulfide bonds between CYS, and the glutenins polymerize into polymers [10]. The formation of disulfide bonds is regulated by the cytosolic and redox conditions in the cell [10]. Furthermore, the level of humidity in the wheat grain is important for the amount and size distribution of the polymers [11]. The ER has been shown to administrate the folding of the proteins, while smaller proteins, such as chaperons and foldases, assist in the folding process [12,13,14]. The proteins are stored at different locations in the plant seed cell in the form of protein bodies (PB) [15], where the transport to the destined locations is co-translational [16,17]. Two trafficking routes have been described for proteins leaving the ER: one is via the Golgi apparatus with deposition in PBs of vacuolar origin, and one is in the ER lumen for later fusion into the vacuole [9,18,19]. The reason for the different pathways is not yet fully understood; the same type of protein has been found to use both pathways, and all types of gluten proteins have been found in the different types of PBs [9,15]. However, segregation among the gluten proteins being deposited in various types of PBs has also been reported, with the actual stage of grain development being suggested as one determinant for the transportation pathway [1,18].

The folding and assembling of gliadins and glutenins into monomers and polymers seems to be a predetermined process dictating the formation of the disulfide bonds within the glutenin polymers and gliadin monomers. Among the gliadins (except for the ω-gliadins), this behavior is observed in the specific intramolecular disulfide bonds that are consistently formed between certain conserved CYS [5,20,21,22]. Similarly, the LMW-GS contains several CYS that form specific intramolecular disulfide bonds, being homologous to those of the gliadins [20,22,23]. Additionally, LMW-GS and HMW-GS form intermolecular disulfide bonds, which leads to glutenin polymers. These are formed by the CYS that are unique to LMW-GS and HMW-GS and absent in the gliadins [5,22,23]. Therefore, the consistency of the formation of the disulfide bonds indicates the importance of structural features in the proteins for disulfide binding opportunities.

To the authors’ knowledge, there are no studies available today that explain the reasons for the differences in crosslinking behavior or in-vivo structural properties of gliadins and LMW-GS. The complexity of gluten proteins constantly hampers cellular studies, where issues like the synthesis of multiple types of gluten proteins, rapid polymerization of several types of the proteins, and difficulties in purification [10,23] are major obstacles. Computer simulations that consider the physical and chemical properties of the amino acids in the protein could here be a valuable tool to describe the structures of the proteins [24]. Both gliadins and LMW-GS contain high contents of proline and glutamine [6], which are known to destabilize the protein structure as well as hinder specific conformations [25]. Proteins that lack specific 3D structural conformations in their native state are classified as intrinsically disordered proteins (IDP) [26], and in previous studies, gluten proteins have also been classified as IDPs [27]. In previous work on IDPs from sources other than gluten, computer simulations have been used on both the atomistic and coarse-grained levels to describe structural properties. Most commonly, the simulated results have been compared and validated with experimental results from various methods, including small-angle X-ray scattering (SAXS) [28,29,30,31,32]. For gluten proteins in particular, only a limited number of studies have focused on the use of computational tools to understand their structural behavior [33,34,35,36,37]. At present, no computational simulation studies are available describing the structural differences between gliadins and LMW-GS in their native state.

Here, atomistic and coarse-grained Monte Carlo simulations were used to characterize two wheat storage protein types, gliadins and LMW-GS. The aim was to understand the differences and similarities in structure-dependent behavior between the two proteins, explaining the reason for the formation of disulfide bonds through intra- and intermolecular interactions. Furthermore, a model for the synthesis of the proteins and their folding is proposed.

## 2. Materials and Methods

### 2.1. Protein Amino Acid Sequences Used for Simulations

In the present study, two proteins, an α-gliadin and an LMW-GS, were selected for Monte Carlo simulations, to understand their structural behavior. The amino acid sequences, as well as their charge distribution, are shown in supplementary A, as collected from the UniProt database (https://www.uniprot.org/) under accession numbers Q9ZP09 and P10386, respectively. They contain 268 (α-gliadin) and 288 (LMW-GS) amino acids with a positive net charge of one (α-gliadin) and seven (LMW-GS). Studies have previously been conducted on these two specific proteins, characterizing their existence and primary structures experimentally [38,39,40]. The origins of the two sequences are spelt wheat and spring wheat [38,39,40], resembling typical α-gliadin and M-type LMW-GS primary sequences according to the literature [7,41]. Here, the sequences of the proteins are considered as representative sequences for gliadins and LMW-GS, respectively, due to the close relationship and similarities with most of the gliadins and LMW-GS [6]. In the simulations, the *N*-terminal sections of 20 and 23 amino acids of both proteins were omitted since these amino acids are known to code for a transport peptide that is removed during synthesis [34,42,43].

### 2.2. Models

Two different types of models, one all-atom and one coarse-grained, in combination with Monte Carlo simulations, were applied to model the protein structures, conformational ensembles, and sensitivity to salinity.

The all-atom models were used to estimate the average folding of the proteins, with and without disulfide bonds, using the force field “Lund FF08” with the software “PROFASI” [44,45]. In principle, the all-atom simulations followed the same principles as described by Fagerberg et al. [46], where the interaction potential includes electrostatic interactions between adjacent peptide units, a contribution from the excluded volume, two types of hydrogen bonds, i.e., backbone–backbone bonds and charged side-chain–backbone bonds, as well as effective hydrophobic interactions. All the interactions were assumed to be pairwise additive. To model intramolecular disulfide bonds, the distance between the involved CYS—more precisely, between the sulfur atoms of the thiol groups— was restrained to a mean distance of 2 Å based on previous findings [47,48]. The restraints were present throughout the entire simulations for all intramolecular disulfide bonds. The modeled disulfide bonds were applied between the CYS pairs listed in Table 1, which have previously been experimentally described [20,21,23].

The coarse-grained models were used to evaluate how electrostatic interactions affect the conformational ensemble of the proteins. Here, each amino acid corresponds to one bead, which can be either charged or neutral depending on the primary sequence. For more information about the model, see supplementary B or [28,49].

The all-atom models treat the water, implicitly utilizing hydrogen bonding, excluded volume, and side-chain potential components to describe it, with cutoffs of 4.3–4.5 Å, which correspond to salinity in the range of 450–500 mM. In the coarse-grained model, the salt and water are treated implicitly and regulated by the Debye screening length and the dielectric constant, respectively, utilizing an extended Debye–Hückel potential. Four different salt concentrations corresponding to 10, 80, 500, or 1000 mM were used to evaluate how the conformational ensemble of the proteins is affected by the charged amino acids. Counter-ions were added explicitly to the simulation box to obtain an electroneutral system.

### 2.3. Monte Carlo Parameters

The all-atom systems were studied utilizing Monte Carlo simulations with the Metropolis algorithm. The simulations were performed in the canonical ensemble, meaning that a constant number of particles (N), volume (V), and temperature (T) were used. The model protein was located in a cubic box of length 1500 Å, where periodic boundary conditions (PBC) were applied in all directions. The force field utilizes cutoffs of 4.3 Å for its excluded volume term and 4.5 Å for its hydrogen bond and side-chain components. Five different displacements were allowed: (i) rotation of the whole chain, (ii) translation of the whole chain, (iii) pivot rotation of single backbone angles, (iv) rotation of side-chain angles, and (v) biased Gaussian steps, which is a local move described by Favrin et al. [50]. For these simulations, an annealing procedure was used to accelerate the simulations and to avoid the simulations becoming trapped in local energy minima [51]. Here, ten different temperatures in the range 300–400 K were applied, and a total of 1000 configurations were produced at each temperature at each cycle. For more information, see supplementary C. The protein model was located with a random start conformation in the box, and an initial simulation run composing of 200 annealing cycles resulting in 2·× 10^5^ configurations at 300 K was performed for equilibration purposes. The following production run involved 800 annealing cycles, corresponding to 8 ×·10^5^ configurations at 300 K. Every 10th of the produced configuration was saved for further analysis, to secure the availability of the variation in the data, simultaneously considering data storage capacity (the exact amount of saved configurations for each temperature and model system is presented in supplementary C).

The equilibrium properties of the coarse-grained Monte Carlo simulations were evaluated using the Metropolis algorithm. For this purpose, the integrated Monte Carlo/molecular dynamics/Brownian dynamics simulation package Molsim [52] was used. The simulations were performed in the canonical ensemble. Further simulation details are described in supplementary B.

### 2.4. Analysis

#### 2.4.1. Hydrophobicity

A hydropathy index graph for the amino acids in the protein was produced with the ExPASy tool “Prot Scale” [53] to describe the hydropathy of the amino acids in the peptide chain. The tool was set to produce a value for every individual amino acid residue in the protein sequence, utilizing the Kyte and Doolittle scale with a window size of 15. The window size was used to illustrate patterns for more extensive parts of the proteins. The chosen scale is based on the free energy of the amino acids when they are transferred between the vapor, ethanol, and water phases [54]. Most hydropathy scales provide a similar estimation of the non-polar amino acid properties but can vary greatly when estimating the polar ones, depending on which solvents the amino acids are transferred between [55]. To broaden the analysis of the proteins, the Rose et al. scale [56] was also used, which estimates an amino acid’s tendency to be buried in hydrophobic cores or to be exposed to solvent at protein surfaces.

#### 2.4.2. Radius of Gyration

The *R_g_* value, also referred to as the mean square distance of the center of mass, for the individual obtained configurations from the all-atom and coarse-grained systems were calculated by built-in modules in the simulation packages PROFASI and MOLSIM.

#### 2.4.3. Polymer Scaling Laws, Shape Factor, and Small Angle X-Ray Scattering (SAXS)

The Flory power law was used to describe the proteins’ behavior in the solvent [57]:

(1) $$R_{g}\alpha \;R_{0}N^{\nu }$$

where the *R_g_* is considered proportional to a constant (*R*_0_) and the number of amino acids (raised to the power of *ν*). *R*_0_ is a prefactor value that equals 2 Å, as determined from experiments on other proteins [58,59]. A *ν* close to 1/3 indicates a collapsed protein structure due to a poor solvent, 1/2 indicates theta conditions, and 3/5 indicates good solvent conditions (an expanded “self-avoiding” chain) [59,60].

To further describe the shape, the ratio of the mean-square of the end-to-end distance (*R_ee_*) and mean-square *R_g_* was used as a shape factor (*R_shape_*).

(2) $$\frac{\left\langle R_{ee}^{2}\right\rangle }{\left\langle R_{g}^{2}\right\rangle }=R_{shape}$$

A *R_shape_* equal to 1 represents a globular shape, 6 a self-avoiding random walk (SARW) shape, and 12 an extended rod shape [28]. Here, the distance between the terminal α carbons was considered as the *R_ee_.*

The spherical average and flexibility of the two proteins were described by a wavelength function derived from the SAXS data, depicted in the form of Kratky plots [61]. The SAXS calculations were in turn performed on the all-atom models using a Debye formula in the foXS software [62,63].

#### 2.4.4. Contact Mapping

Contact maps were generated in the all-atom simulations to illustrate the distance between the amino acids. Here, the built-in function module “generate native contact list” in the PROFASI software was used. The module was set to register distances closer than 5 Å between all non-hydrogen atoms in the amino acid side-chains, which resulted in distinguishable patterns of the protein contacts. The contact map does not register distances between adjacent amino acids.

#### 2.4.5. Secondary Structure Estimation

The software STRIDE [64], which estimates secondary structure based on patterns of hydrogen bonding and backbone angles, was used to estimate the secondary structure for each conformation of the all-atom simulations.

#### 2.4.6. Plotting, Graphics, and Statistical Calculations

All plotting and statistical calculations were made with the statistical programming language R version 3.5.1, “Feather Spray” [65]. The packages used for plotting purposes were as follows: ggplot2, scales, egg, gridExtra, grid, plyr, dplyr, doParallel, ggraph, ggpubr, and igraph.

The 3D rendering graphics were made with the molecular visualization software VMD, with tachyon in-memory rendering settings [66].

## 3. Results

### 3.1. Shape Classification Based on Charges

**Figure 1:**
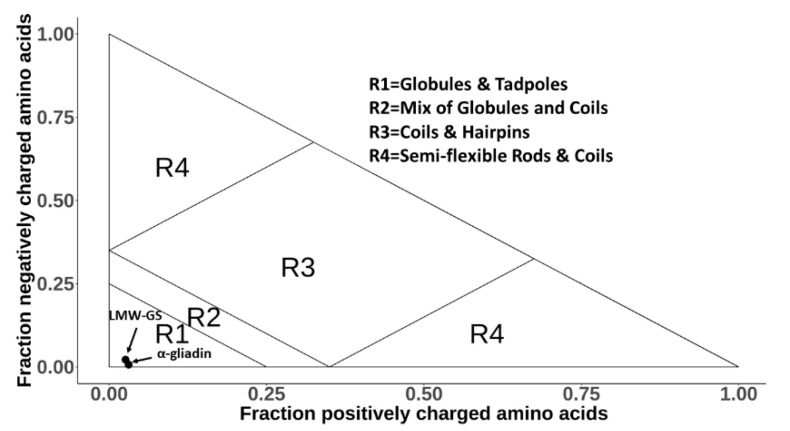
Das–Pappu plot, where intrinsically disordered proteins (IDPs) are categorized into four different structure categories depending on their fractions of charged amino acids [67,68]. Both the α-gliadin and the LMW-GS are positioned in the R1 category, meaning that their shape is supposed to be either globular or tadpole-like.

At physiological pH, the α-gliadin carries seven positive and six negative charges and the LMW-GS nine positive and two negative charges, resulting in corresponding net charges of +1 and +7. This would suggest that the proteins are classified as polar tracts with globular and tadpole shapes according to the Das–Pappu classification (Figure 1) [67,68]. However, the prediction is uncertain due to a high content of proline and hydrophobic amino acids which, in the Das–Pappu classification, have an unknown impact on protein structure.

### 3.2. Hydrophobicity of Gluten Proteins

**Figure 2:**
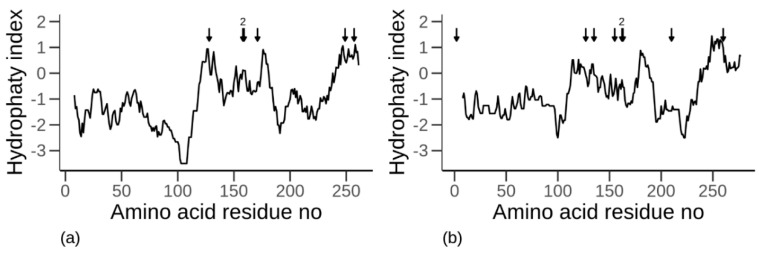
Hydropathy index (hydrophobicity for each amino acid with a window size of 15 amino acids) for each amino acid residue of (**a**) α-gliadin and (**b**) low molecular weight glutenin, indicating hydrophobic (+) and hydrophilic (−) regions in the protein. Cysteine amino acids are marked with an arrow, and the number 2 indicates two vicinal cysteines marked with two arrows at that position.

Differences in hydropathy were observed along the amino acid chain for both α-gliadin (Figure 2a) and LMW-GS (Figure 2b) after the primary sequences were evaluated against the Kyte and Doolittle scale with the Prot Scale tool. In general, the proteins were similar from a hydropathy perspective, although some differences were visible between the two proteins (compare Figure 2a with Figure 2b). The segments containing the first 100 residues of amino acids were similar for the α-gliadin and LMW-GS, being rather hydrophilic, although with some parts of lower hydrophilicity and also a larger variation in hydrophilicity in the α-gliadin (Figure 2a) as compared to the LMW-GS (Figure 2b). A short, highly hydrophilic segment was found in both proteins directly after amino acid residue 100. Furthermore, for both proteins, the segments from amino acid residue 125 to approximately amino acid residue 180 were found to be more hydrophobic, but again with more hydrophilic parts in α-gliadin compared to LMW-GS. In the next segment, from approximately amino acid residue 180 to 250, both proteins were found to be rather hydrophilic, with a slightly higher amount of hydrophilicity for the LMW-GS than α-gliadin. Then, from amino acid residue 250 to the end of the protein, both proteins were again found to be relatively hydrophobic. The accumulated hydropathy index for α-gliadin was −0.98 (variation of 1.08), whereas the corresponding number for LMW-GS was −0.71 (variation of 0.75). The Rose et al. [56] scale provided a similar depiction of the proteins (supplementary D).

The positions of the CYS in each of the proteins (Figure 2) indicate that all CYS, including the vicinal ones, are located in relatively hydrophobic regions (−0.5 to +1) for the α-gliadin (Figure 2a). This is contrary to the position of two of the CYS (1 and 7 (from left to right) of the CYS in LMW-GS, Figure 2b) in the LMW-GS that are located in relatively hydrophilic regions (−1 to −1.5). The remaining CYS (2–6 and 8) in the LMW-GS were located in the hydrophobic regions of the protein, similarly to the CYS in the α-gliadin (Figure 2b)

### 3.3. Protein Solubility and Salt Effects

**Figure 3:**
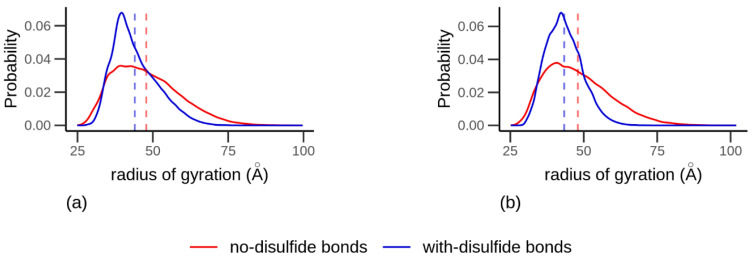
The probability distribution of the radius of gyration for (**a**) α-gliadin and (**b**) low molecular weight glutenin from all-atom simulations, where the red curve indicates a protein without intramolecular disulfide bonds, and the blue curve indicates a protein with intramolecular disulfide bonds according to experimental data [20,21,23]. The broken line displays the mean of the curve.

The probability distribution of *R_g_* (Figure 3) for the all-atom simulations was skewed for both proteins without disulfide bonds, with a mean of 47.8 Å and 47.9 Å and with a variance of 118 Å and 117 Å for α-gliadin and LMW-GS, respectively, indicating an open structure. A less skewed distribution, with mean values of 44.0 Å and 43.3 Å and a variance of 55 Å and 36.5 Å, respectively, was found for α-gliadin and LMW-GS with intramolecular disulfide bonds, indicating a more compact structure. Intramolecular disulfide bonds in the proteins also contributed to larger peaks and a close to normal probability distribution function in terms of *R_g_*, the latter especially for the LMW-GS protein.

The *R_g_* for the coarse-grained models did not alter with variations in salinity and provided a root square mean (rms) of 63 Å for α-gliadin and 58 Å for LMW-GS, results which are compared with the all-atom model results in supplementary B. The small change in *R_g_* due to altered Debye screening length and the small energy contributions indicate that the proteins are composed of a relatively low amount of charged amino acids (net charge +1 and +7), shown in the charge distribution map in supplementary A, with low impact on the proteins’ conformation.

Flory values (*ν*) of 0.55 and 0.57 were obtained with and without intramolecular disulfide bonds, respectively, for both the all-atom α-gliadin and LMW-GS models when applying the Flory scaling law; thus, the implicitly modeled water can be considered as a theta solvent for both proteins, since *ν* is between 1/2 and 3/5.

### 3.4. Protein Shape and Internal Structural Arrangement

**Figure 4:**
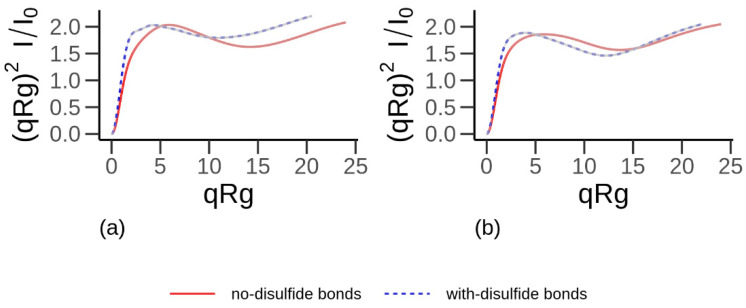
Dimensionless Kratky plot (visualizing shape of proteins) of α-gliadin (**a**) and low molecular weight glutenin (**b**). The solid red curve corresponds to protein without intramolecular disulfide bonds, and the dashed blue curve corresponds to proteins with intramolecular disulfide bonds. Data obtained from all-atom simulations. *q* is the scattering vector [61] (also referred to as the momentum transfer), *I* is intensity, and *I*_0_ is the intensity of the incoming ray.

The Kratky plots of the α-gliadin (Figure 4a) and the LMW-GS (Figure 4b) from the all-atom simulations visualized the similarity in the folding of the two proteins. Both proteins showed a broad maximum at *qR_g_* of 1 to 12, indicating the presence of slightly denser or more folded regions in the proteins. For 12 < *qR_g_* < 15, the curves were relatively horizontal, indicating the presence of unfolded regions in both proteins. Thereafter, a slight increase is shown, indicating that both proteins also contain extended regions. When proteins were modeled with the proposed disulfide bonds [20,21,22,23], the Kratky plots resulted in a maximum/shoulder occurring at lower *qR_g_* values, indicating both proteins to be denser or more folded in comparison to the case without disulfide bonds.

The calculations of the estimated shape of α-gliadin and LMW-GS yielded the following averaged *R*_shape_ values: when disulfide bonded, 2.97 and 2.18, respectively, and when not disulfide bonded, 4.9 and 5.1, respectively. The distribution of the *R*_shape_ values can be found in supplementary E. Thus, these calculations indicated a shape in-between globular and SARW, with a more globular shape when disulfide bonds are present.

**Figure 5:**
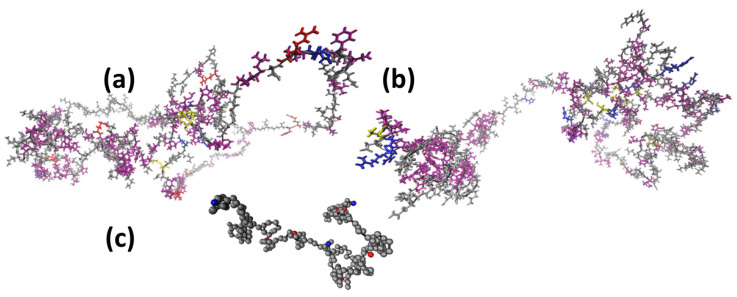
Simulation snapshots of all-atom (**a**) α-gliadin, (**b**) LMW-GS, and coarse-grained (**c**) α-gliadin. The grey colored groups represent neutral amino acids/beads, red indicates positively charged, blue negatively charged, purple are amino acids with hydrophobic potentials, and yellow are cysteine amino acids.

Ring and tail like motifs could often be observed when visually inspecting snapshots from all-atom simulations of both proteins when these contained disulfide bonds (Figure 5a,b). The ring in the present snapshots is located between CYS 171 (being from amino acid residue 171 of the protein) to CYS 249 in α-gliadin and LMW-GS between CYS 163 to CYS 260. The tail in the present snapshots is observed from the *N*-terminal to the vicinity of amino acid 120 in both proteins, yielding a tadpole-like structure. The proteins had random chain structures when similar visual inspections were performed on the coarse-grained simulated structures (Figure 5c).

**Figure 6:**
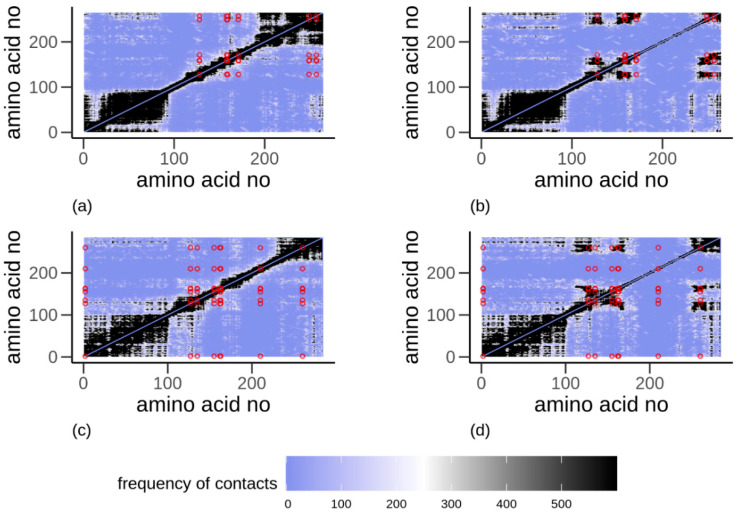
Contact maps for (**a**,**b**) α-gliadin and (**c**,**d**) low molecular weight glutenin, (**a**,**c**) without disulfide bonds and (**b**,**d**) with disulfide bonds obtained from all-atom simulations. Dark regions indicate a high amount of contacts among amino acids. A high degree of contact among amino acid residues positioned two steps away is seen as a black diagonal. Dark areas annotated by circles show a high degree of contact between disulfide bonded cysteine residues.

Additionally, the contact maps for the α-gliadin (Figure 6a,b) and the LMW-GS (Figure 6c,d), determined with (Figure 6b,d) and without (Figure 6a,c) disulfide bonds, indicated high similarity in the protein structure between the two evaluated proteins. An increased contact among amino acids for the next-nearest neighbors is visible for both proteins, independently of whether they are disulfide bonded or not; see black diagonal (Figure 6a–d). Furthermore, both proteins, with or without disulfide bonds, show a higher probability in the *N*-terminal and the repetitive region (residue 1 to 100), i.e., enhanced contacts. Differences among the evaluated proteins are visible in the C-terminal region of the proteins, where the α-gliadin shows an increased probability of contact from residue 200 and onward, whereas the LMW-GS shows contacts of amino acids primarily from residue 250 and onward. For disulfide-bonded proteins, only a low frequency of contacts is found in the C-terminal end for both proteins. Instead, distinct areas of contacts between amino acids increase in the vicinities of the CYS involved in the disulfide bonds.

**Figure 7:**
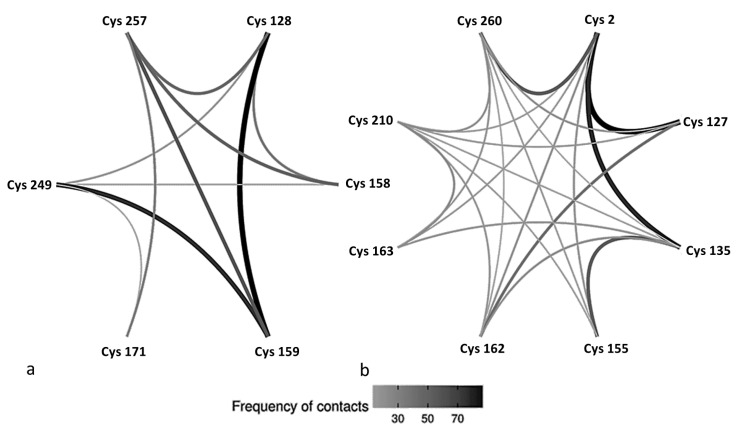
Contact map between cysteine residues in (**a**) α-gliadin without disulfide bonds and (**b**) low molecular weight glutenin without disulfide bonds. The darker color of the line corresponds to a higher frequency of contacts. The results are derived from all-atom simulations.

Determination of the frequency of contacts between various CYS for the proteins without disulfide-bonds indicated that all residues are occasionally in contact with other CYS (not necessarily all other CYS) (Figure 7) during the all-atom simulation. For α-gliadin, CYS 159 showed an elevated occurrence with three CYS (CYS 128, CYS 249, and CYS 257). The other CYS in α-gliadin showed different tendencies for contacts. For the LMW glutenin, CYS 2 and CYS 210 showed contacts with all other cysteine residues in the proteins, although, for CYS 210, the instances of contact were generally low, in contrast to CYS 2, which had a high number of contacts with CYS 127, Cys 135, and CYS 260. A relatively high frequency of contacts was also visible between CYS 127 and CYS 162, as well as between CYS 135 and CYS 155. Since the software does not register contacts for adjacent amino acids, there are no data on the frequency of contacts for the vicinal CYS (CYS 158 and 159 in α-gliadin or CYS 162 and 163 in LMW-GS).

### 3.5. Secondary Structure

**Figure 8:**
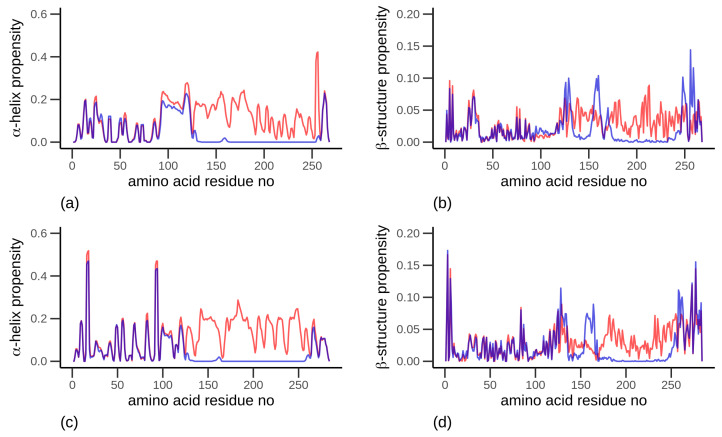
Propensity of secondary structure for (**a**,**c**) α-helix and (**b**,**d**) β-sheets/strands, for (**a**,**b**) α-gliadin and (**c**,**d**) low molecular weight glutenin. The red curves indicate proteins without intramolecular disulfide bonds, and the blue curves indicate protein with intramolecular disulfide bonds. Observe the differences in scales, which were used to more clearly visualize the β-structure propensity. The data is obtained from all-atom simulations.

The α-gliadin (Figure 8a,b) and the LMW-GS (Figure 8c,d) showed a similar secondary structure propensity in the absence of intramolecular disulfide bonds (dark curve), with a relatively uniform distribution of α-helices and β-sheets/strands, whereas when intramolecular disulfide bonds are included, there is an increased tendency for β-sheets/strands for the amino acids involved in these specific bonds. In general, the proteins showed a large change in secondary structure propensity when disulfide bonds were introduced (compare dark-grey curves with light-grey curves in Figure 8), resulting in a diminishing of α-helices (from around amino acid residue 125 and onwards). In contrast, for amino acid residues 1–125, no such effects were visible.

## 4. Discussion

The present study clearly demonstrates the striking similarities between two of the main types of wheat grain storage proteins, gliadins and LMW-GS, concerning secondary structure, folding, size, and shape, according to the simulated results. The main difference between the two proteins was the presence of two CYS residues located in a relatively hydrophilic part of the LMW-GS, while all other CYS residues, both in the α-gliadin and in the LMW-GS, were located in more hydrophobic parts of the molecules. The two CYS located in the more hydrophilic part of the LMW-GS form intermolecular disulfide bonds, while the other CYS in both proteins forms intramolecular disulfide bonds [20,22].

Earlier studies of wheat storage proteins classified LMW-GS as an aggregated type of gliadin, which was corrected in the 1980s by Shewry and co-workers [6,69,70,71]. Since then, additional similarities have been defined between the two proteins within their primary structures, i.e., amino acid repeats, high level of proline and glutamine, vicinal CYS, etc. [7]. Such similarities have led to the conclusion that gliadins and LMW-GS share a common evolutionary ancestor [72]. Recent genomic studies on the gliadin and LMW-GS loci have elucidated a great extent of repetitive DNA and genes with tandem duplication, thereby challenging sequence assembly studies [73]. Sequencing and analyses of orthologous regions of gliadin and LMW-GS genes have shown relatively large differences between these gene families. The LMW-GS genes were found to be less clustered and separated by more considerable distances, due to the insertion of repetitive blocks and interspread gene loci, as compared to the gliadin genes [74]. Besides primary structure, structural features of the gliadins and the LMW-GS have had limited description, basically due to the aggregation and intrinsically disordered features of the proteins complicating such studies [24]. Therefore, the present study is one of the first of its kind to contribute to describing the similarities in terms of secondary structure, hydropathy, and folding patterns based on Kratky plots and contact maps for both protein types.

In the present study, it was possible to describe some of the potential background reasons and mechanisms behind the formation of intramolecular disulfide bonds within both proteins. The formation of intramolecular disulfide bonds within both proteins is specific, with particular CYS always forming bonds with each other [5,20,21,22]. Certain intramolecular disulfide bonds may also affect the formation of other intramolecular disulfide bonds, e.g., the bond between CYS 127 and 162 in LMW-GS may prevent the protein from forming aberrant aggregates [75,76]. Our results indicate that the hydrophobic amino acid segments, adjacent to the CYS involved in intramolecular disulfide bonds, are probably crucial for disulfide bond formation, as also reported for elastin aggregation [77]. Until now, the impact of hydrophobicity in the amino acid chain of the gluten proteins on the formation of crosslinks in the wheat seed has been limitedly experimentally studied. Systematic mutations changing this property along the amino acid chain is an interesting idea for a future study on the topic that would allow a more in-depth understanding of its effect on crosslinking. However, such studies of wheat grain are still challenging to perform experimentally. Moreover, by simulations, where the mutations can easily be incorporated in the amino acid chain, modeling tools need to be developed to be able to describe the effects on crosslinking by the changed hydrophobicity. Furthermore, necessary experimental verifications are also still lacking.

Further describing the potential crosslinking backgrounds for the two proteins, previous studies have indicated that the CYS crosslinking of the LMW-GS takes place as a polymerization directly after the formation of the proteins in the ribosomes of rough ER and transportation to ER lumen [10]. Our results regarding contacts between CYS within the gliadin and LMW-GS molecules, respectively, indicate flexibility; hence, most of the CYS have an opportunity to reach each other for the formation of disulfide bond crosslinks. However, one can presume that the intramolecular disulfide bonds are not formed simultaneously; a bond is likely to be formed immediately after CYS is produced in an intramolecular disulfide bond couple. This rapid chronological bond formation results in intramolecular disulfide bonds, in accordance with previous reports [5,20,21,22]. Thus, the intramolecular disulfide bond formation can be seen to occur based on the chronological position of CYS and predicted contact propensity for CYS presented in this study, i.e., in α-gliadin, CYS 127 is synthesized first and has frequent contact with the second synthesized CYS 158, where a disulfide bond is formed. Then, CYS 159 is next synthesized and has contacts with the fifth synthesized CYS 249 and forms a disulfide bond, since there is already a bond present between CYS 127 and 158, and so on.

Two of the LMW-GS CYS (CYS 2 and CYS 210) are, according to previous studies, responsible for the formation of intermolecular disulfide bonds [5,22,23]. Following the discussion above, neither CYS 2 nor CYS 210 form intramolecular disulfide bonds due to the lack of neighboring hydrophobic amino acids. The present study clearly shows that CYS 2 is located in a mobile part of the molecule, as shown by a large number of contacts with other CYS in the contact map evaluation. In contrast, CYS 210 is located in a more rigid part of the molecule, as seen from the contact map evaluation. As intermolecular disulfide bonds are most likely also formed directly after synthesis or during synthesis, the N-terminal CYS (CYS 2) crosslinks with surrounding GS at an earlier stage than CYS 210, as has been described using in-vitro studies of LMW-GS proteins [10]. The C-terminal CYS (CYS 210) is more passive, with a slower rate of crosslinking, corresponding to previously reported results [75]. The CYS 210 part of the protein is also more likely transformed into α-helical or β-strand/sheet structures before the intermolecular disulfide bonds are formed, in line with the secondary structure propensity results from this study. In addition to the described crosslinking steps, disulfide genomic differences, with different distances between encoding genes, have also been described as one possible contributor to the differences in intra/intermolecular disulfide bond formation [74].

Intermolecular disulfide bond formation is the basis by which the gluten proteins form their large polymers and involves, together with the LMW-GS, the HMW-GS [20,23]. Intermolecular disulfide bond formation, like intramolecular disulfide bond formation, also seems to be a rather pre-determined event, with certain CYS at particular GS proteins more commonly crosslinking to each other [7,20,23]. Previous theoretical models have described the directionality of the formation of the polymers (head-to-tail, head-to-head, tail-to-tail) [78], which also indicate the pre-determined formation of the polymers. Determination of the molecular background factors of the intermolecular disulfide bond formation were not the aim of this study, but features described here for the LMW-GS—e.g., hydropathy, time, and chronology of synthesis of the CYS; flexibility of the amino acid chain; structure formation—might play a role in determining the crosslinking. Similarly, as was found for the LMW-GS in the present study, the crosslinking sites of the HMW-GS have been reported to be located in hydrophilic regions of the proteins [79,80]. Moreover, recent investigations have indicted surface hydrophobicity in the *N*-terminal domains of HMW-GS as an important factor for interdisulfide bond formation [81].

The α-gliadin model applied in the present study corresponded well with previous experimental data on the protein. The α-gliadin has, in correspondence with simulations in the present study, been experimentally characterized with a similar size, secondary structure, and lack of a particular fold conformation and is described as a disordered protein with a secondary structure mostly occupied by random coils and turns [82,83,84,85,86]. In particular, previous work determines the size of α-gliadin *R_g_* to be between 35.5 and 41 Å [84,86], which is within the *R_g_* distribution range mostly populated by the simulations. Previous studies [84,86] have described the protein to fit an elliptical model, while here, the protein shapes correspond to an intermediate structure between a globular and expanded SARW shape, possibly an elliptical shape. These potential model differences may be related to force field, solvent type, and/or concentrations of proteins and solvents applied. To our knowledge, similar experimental data as for the gliadin, describing the native monomeric “wild-type” LMW-GS before aggregation, is not available in the literature. This lack of data may be the result of the extensive aggregation through disulfide crosslinking that the protein undergoes directly after/during synthesis [10,76], suggesting that there are no obtainable monomeric native LMW-GS. Only in a few studies, the structural features have been investigated of more hydrophobic IDPs, although those indicate that these proteins are expanded or in theta conditions in an aqueous environment [87], which is in line with the results presented here. Furthermore, polyampholyte and polyelectrolyte IDPs can also have an expanded shape [67,68]. However, neither α-gliadin nor LMW-GS can be classified as polyampholytes or polyelectrolytes since the charged amino acids had only a minor effect on the model structures presented here.

**Figure 9:**
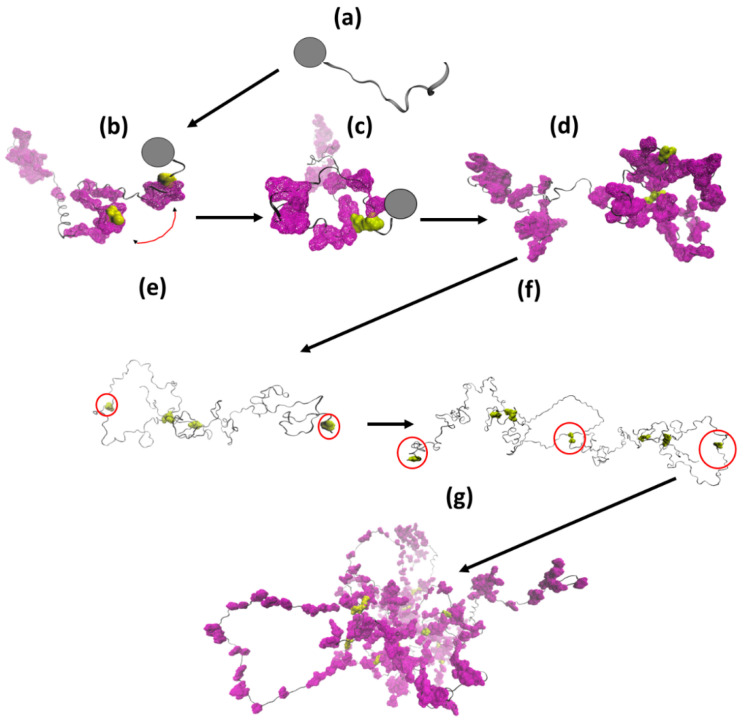
A proposed model for the synthesis, folding, disulfide bond formation, and storage of α-gliadin and low molecular weight glutenin subunits in the wheat grain: (**a**) start of the synthesis of the α-gliadin at the ribosome (dark circle); (**b**) as soon as cysteines residues (yellow surfaces) are synthesized, adjacent sections of amino acids influence the 3D-shape of the molecule, and those which are hydrophobic (purple surfaces) bring cysteine residues close; (**c**) closeness of cysteine residues contributes to the formation of intramolecular disulfide bonds (yellow areas); (**d**) a fully synthesized α-gliadin with disulfide bonded cysteine residues (yellow areas) and a disordered fold; (**e**) a fully synthesized LMW-GS that has undergone the same formation of intramolecular disulfide bonds (yellow areas) but with two cysteine residues (with neighboring sections of amino acids that are less hydrophobic) that are not crosslinked (here encircled in red); (**f**) intermolecular disulfide bonds are formed after protein synthesis for the LMW-GS; (**g**) protein bodies are formed by glutenins and gliadins through intermolecular disulfide bond crosslinks (LMW-GS) and hydrophobic interactions.

The present study investigated the structural properties of one α-gliadin and one LMW-GS, chosen as good representatives of the gliadin and LMW-GS types of storage proteins in wheat. Despite the rather high level of diversity within the two protein type groups, we hypothesize enough similarities between different proteins within these groups to draw general conclusions based on the results of the present study. Based on the results in the present study and previous findings described above, we propose a model describing the synthesis and folding of the two wheat storage proteins, the gliadins and the LMW-GS, and their assembly into protein bodies (Figure 9). The proteins are synthesized in the cell at the rough ER (Figure 9a), where folding occurs, and intramolecular disulfide bonds are formed. The hydrophobicity of the amino acid peptide sections that are next to the CYS regulates the predetermined intramolecular disulfide bonds to be formed. Thus, the intramolecular disulfide bonds of the protein are formed sequentially and rapidly after each associated CYS is synthesized (Figure 9b,c). After this, the LMW-GS forms intermolecular disulfide bonds (Figure 9e,f), starting with the formation of bonds involving the CYS present in the more flexible *N*-terminal of the protein. The CYS forming intermolecular disulfide bonds are located next to amino acid peptide sections that are less hydrophobic than those close to CYS forming intramolecular disulfide bonds. This is the reason that glutenin polymerization is affected by cytosolic conditions in the cell, e.g., redox conditions. Since external conditions such as humidity and precipitation affect the cytosolic conditions in the cell, the cellular location in which the polymerization occurs explains differences in the size distribution of storage protein polymers in wheat. Such variation in polymer distribution is known to impact wheat quality [88,89]. Additionally, other external conditions, such as temperature, drought, and plant development time, to mention a few factors, are known to affect wheat storage protein polymerization during grain maturation, and maturity impacts the conditions of the cell both directly and indirectly by differing plant responses [8,90]. Chaperons and foldases might assist in the folding process of the storage proteins, although this has not been evaluated by us, and the results of this study indicate good opportunities for the correct folding and formation of disulfide bonds without such contributions. However, when forming protein bodies, chaperons and foldases might provide functions for correct packing and transport signaling, which has been described for homologous storage proteins in other grass species [14,91]. The formation of protein bodies is most probably a result of similar hydrophobic interactions and crosslinking, as described above (Figure 9g). An increasing concentration and amount of hydrophobic groups contribute to proteins more prone to self-associating [77,92], which is speculated to be an initial stage of protein body formation for gliadins [93,94]. Folded and aggregated proteins are then transported via different routes to the assembly in PBs at various places in the cell, depending on the stages of grain development.

## 5. Conclusions

Our models of gliadins and LMW-GS are strikingly similar when it comes to secondary structure, size, and shape. The major difference between the two proteins are two CYS in the LMW-GS, positioned in parts of the protein with lower hydrophobicity compared to the rest of the CYS in the LMW-GS and also all CYS in the gliadins. These two CYS form intermolecular disulfide bonds, while the rest of the CYS residues form intramolecular disulfide bonds. The hydrophobicity of the adjacent section amino acids, the chronology of synthesis of the amino acids, and the flexibility of the amino acid chain for the contact between CYS to occur are the major determinants for when and how different intra- and intermolecular disulfide bonds are formed, following a predetermined structure. Generally, intramolecular disulfide bonds are formed first, among CYS with adjacent sections of hydrophobic amino acids, and in the order of synthesis, if allowed by amino acid chain flexibility. After this, intermolecular bonds form the polymers, where CYS in the mobile part of the amino acid chain plays a more active role than CYS in the more rigid part of the protein. Cytosolic conditions influence the rate and complexity of intermolecular disulfide bonds formed.

## Figures and Tables

**Table 1:** Disulfide bond pairs in α-gliadin and low molecular weight glutenin subunits (LMW-GS), where the cysteines (CYS) number refers to the CYS amino acid position.

α-gliadin	LMW-GS
CYS 128–CYS 158	CYS 2—Interdisulfide bond
CYS 159–CYS 249	CYS 127–CYS 162
CYS 171–CYS 257	CYS 135–CYS 155
	CYS 163–CYS 260
	CYS 210—Interdisulfide bond

## Supplementary Materials

The following are available online at https://www.mdpi.com/2218-273X/10/8/1095/s1. References [95,96] are cited in the supplementary materials.
