# Supplementary material for: Glutenin and Gliadin, a Piece in the Puzzle of their Structural Properties in the Cell Described through Monte Carlo Simulations
Source: Biomolecules. 2020 Jul 23;10(8):1095. doi: 10.3390/biom10081095 (PMC7465137; doi:10.3390/biom10081095)
Supplement: Supplementary file 1 [file biomolecules-10-01095-s001.zip › supplementary/Supplementary.docx]

Supplementary A

The primary structure for the α-gliadin used, the sequence in brackets belongs to signal peptide not used in this study:

“(MKTFLILALLAIVATTATTA)VRVPVPQLQPQNPSQQQPQEQVPLVQQQQFLGQQQPFP

PQQPYPQPQPFPSQQPYLQLQPFPQPQLPYSQPQPFRPQQPYPQPQPQYSQPQQPISQQQQ

QQQQQQQQQQQQQQQQILQQILQQQLIPCMDVVLQQHNIAHGRSQVLQQSTYQLLQELCCQ

HLWQIPEQSQCQAIHKVVHAIILHQQQKQQQQPSSQVSFQQPLQQYPLGQGSFRPSQQNPQ

AQGSVQPQQLPQFEEIRNLALQTLPAMCNVYIPPYCTITPFGIFGTN”

Charge distribution map for the α-gliadin, where + refers to positively charged, – negatively charged and · neutral amino acids:

“(·+··················)·+·················-················

······································+·····················

·································-···········+·············-

··········-········+···········+·························+

····················—·+······························”

Net charge for α-gliadin is + 1.

The primary structure for the LMW-GS with the same notations as for the α-gliadin:

“(MKTFLVFALLAVAATSAIAQMET)RCIPGLERPWQQQPLPPQQTFPQQPLFSQQQQQQL

FPQQPSFSQQQPPFWQQQPPFSQQQPILPQQPPFSQQQQLVLPQQPPFSQQQQPVLPPQQS

PFPQQQQQHQQLVQQQIPVVQPSILQQLNPCKVFLQQQCSPVAMPQRLARSQMLQQSSCHV

MQQQCCQQLPQIPQQSRYEAIRAIIYSIILQEQQQVQGSIQSQQQQPQQLGQCVSQPQQQ

SQQQLGQQPQQQQLAQGTFLQPHQIAQLEVMTSIALRILPTMCSVNVPLYRTTTSVPFGV

GTGVGAY”

Charge distribution map for the LMW-GS:

“(·+·····················)+·····-+·························

·····························································

·································+··············+··+·······

····················+·-··+·································

·······································+·············+······

··········”

Net charge for LMW-GS is +7.

Supplementary B

Supplementary B. 1 Model description

In the coarse-grained model [28,49], the amino acids of the proteins are represented by hard spheres (beads) that mimic their excluded volume including the hydration layer, and connected via harmonic bonds. The N- and C-termini are treated explicitly to account for the extra charge of the protein terminals. The bead radius was set to 2 Å providing a realistic contact separation between the charges and accurate Coulomb interaction. The non-bonded spheres interact through a short-ranged attractive interaction as well as electrostatic interactions, where the interparticle electrostatic interactions are described on the Debye–Hückel level. The simulations are performed at constant pH with point charges. Each monomer is either negative, positive, or neutral, depending on the amino acid. All values except the ones for the selected screening lengths are from the work of Cragnell et al. [28], which describes the characteristics of IDPs.

The total potential energy of the system U_total_ contains bonded and non-bonded contributions and is given by:

| $U_{total}=U_{non-bonded}+U_{bonded}=U_{hs}+U_{el}+U_{short}+U_{bonded},$ | (1) |
| --- | --- |

The hard-sphere potential (*U_hs_*) is defined as:

| $U_{hs}=\sum_{i,j,i\neq j} u_{ij}^{hs}\left( r_{ij} \right),$ | (2) |
| --- | --- |

where the distance between particle pairs (r_ij_) is the centre-to-centre distance. The excluded volume of the amino acid is taken into account through the hard-sphere potential, $u_{ij}^{hs}$ is given by:

| $u_{ij}^{hs}\left( r_{ij} \right)=\left\{ \begin{aligned} 0, \\ \infty, \end{aligned}\begin{matrix} r_{ij} \\ r_{ij} \end{matrix}\begin{matrix} {>R}_{i}+R_{j} \\ {<R}_{i}+R_{j} \end{matrix} \right.,$ | (3) |
| --- | --- |

where *R_i_* and *R_j_* are the radii of the beads. The electrostatic interactions U_el_ are handled through an extended Debye-Hückel potential given by:

| $U_{el}= \sum_{ij} u_{ij}^{el}r_{(ij)}=\sum_{i<j} \frac{Z_{i}Z_{j}e^{2}\exp\left[ -k\left( r_{ij}-\left( R_{i}-R_{j} \right) \right) \right]}{{4\varepsilon}_{0}\varepsilon_{r}\left( 1+kR_{i} \right)\left( 1+kR_{j} \right)r_{ij}},$ | (4) |
| --- | --- |

where *e* is the strength and Z (positive, negative or neutral) is the type of the elementary charge, *k* is the inverse Debye screening length, $\varepsilon_{0}$ is the vacuum permittivity, and $\varepsilon_{r}$ is the dielectric constant for water 78.4 [95]. The inverse Debye screening is used for salinity:

| $k^{-1}=\sqrt{\frac{\varepsilon_{0}\varepsilon_{r}k_{B}T}{2N_{A}e^{2}I}},$ | (5) |
| --- | --- |

where *k_B_* is the Boltzmann constant, *N_A_* is Avogadro’s number, and *I* is the ionic strength, which in this study was set to 10, 80, 500 or 1000 mM.

The short-ranged attractive interaction between the monomers is included through an approximate arithmetic average overall amino acids, given by:

| $U_{short}=-\sum_{i<j} \frac{\varepsilon}{r^{6}},$ | (6) |
| --- | --- |

where $\varepsilon$ was set to 0.6·10^4^ kJÅ^6^/mol, based on the work by Cragnell et al. [28], and sets the strength of the interactions based on the polarizability of the proteins. In this model, ε provides an attractive potential of 0.6 kT at closest contact.

The beads in the protein are connected by a harmonic bond potential, U_bonded_:

| $U_{bonded}=\sum_{i=1}^{N-1} \frac{k_{bond}}{2}\left( r_{i,i+1}-r_{0} \right)^{2},$ | (7) |
| --- | --- |

where *r_i,i_*_+1_ is the distance between two connected beads, *r*_0_ with a value of 4.1 Å is the equilibrium distance, and *k_bond_* with a value of 0.4 N/m describes the spring force constant, based on the work by Cragnell et al. [28].

The proteins are assumed to be flexible, where only the electrostatic interactions between the beads, as well as the volume of the hard spheres, contribute to the rigidity of the protein.

Supplementary B. 2 Monte Carlo Parameters

The coarse-grained protein model chain was inserted in a cubic box of length 1500 Å where periodic boundary conditions (PBC) were applied in all directions. The long-range Coulomb interactions were truncated using the minimum image convention at a cut-off length of 750 Å. Four different types of displacements were allowed: (i) translational displacement of a single bead, (ii) pivot rotation, (iii) translation of the entire chain, and (iv) slithering move, in order to accelerate the examination of the configurational space [96]. The probability of the different trial moves was weighted to enable single particle moves 20 times more often than the other three. Initially, the protein was randomly located in the box, and an initial equilibrium simulation of typical 2·10^5^ trial moves/bead was performed for equilibration purposes, whereas the proceeding production run comprised 2·10^6^ passes divided into 10 subdivisions. To confirm that the simulations were sampled accurately, the radius of gyration (*R_g_*) probability distribution functions were analyzed. The uncertainty of the *R_g_* values is based on the standard deviation of the total mean and the mean from the ten subdivisions, as described in Cragnell et al. [28].

Supplementary B. 3 Results


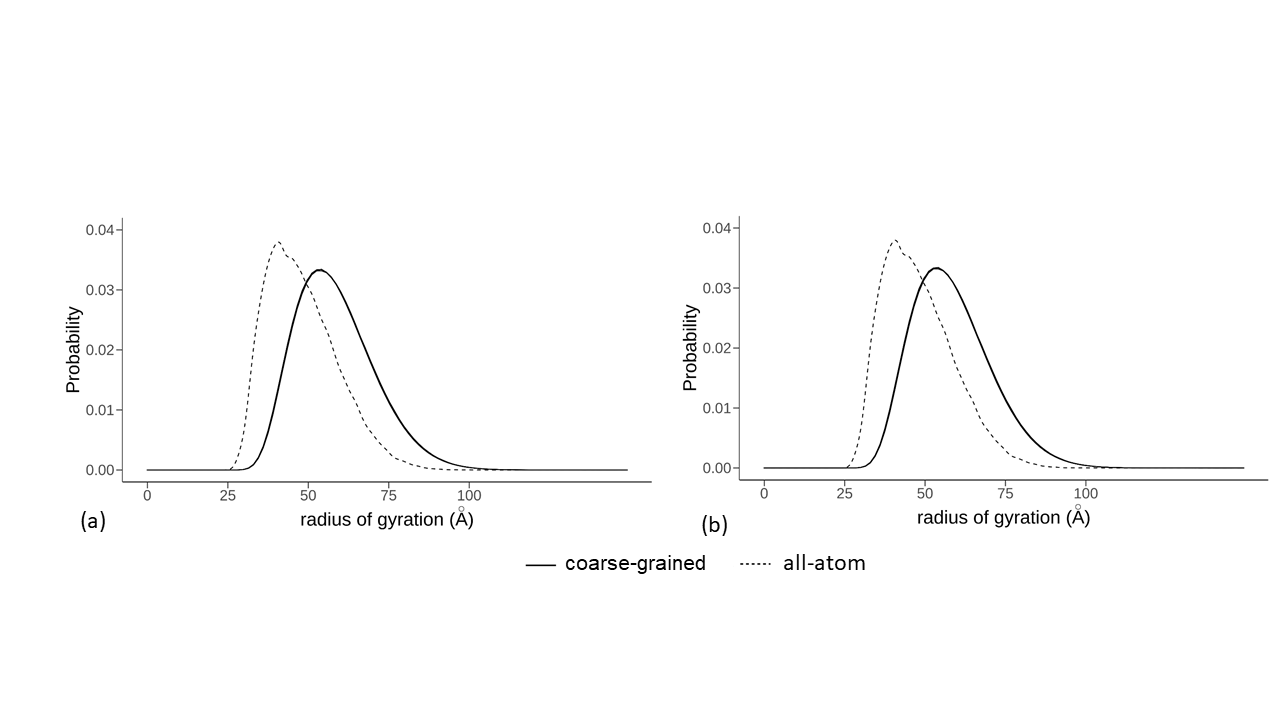


**Figure 10**. Probability distribution comparison between coarse-grained (solid line), and all-atom without disulphide bonds, dashed line, simulations for (**a**) α-gliadin and (**b**) low-molecular-weight glutenin. The coarse-grained simulations are four plotted curves representing the results from the salt treatment, they are perceived as one curve due to their high similarities.

The *R_g_* distribution from the coarse-grained models shows that the values are larger in comparison to the all-atom simulations without disulphide bonds (Figure 10). Both types of simulation techniques describe that α-gliadin and LMW-GS are similar in terms of *R_g_*.

Supplementary C

**Table 2.** Amount of collected configurations for each all-atom model at the corresponding temperature.

| **Temperature** | **α-gliadin with disulphide bonds** | **α-gliadin without disulphide bonds** | **LMW-GS with disulphide bonds** | **LMW-GS without disulphide bonds** |
| --- | --- | --- | --- | --- |
| 300 | 80087 | 80000 | 80000 | 80088 |
| 309.74 | 80100 | 80066 | 80066 | 80286 |
| 319.8 | 80125 | 80100 | 80170 | 80500 |
| 330.2 | 80238 | 80206 | 80377 | 80581 |
| 340.9 | 80369 | 80415 | 80400 | 80633 |
| 351.99 | 80528 | 80544 | 80504 | 80720 |
| 363.42 | 80600 | 80600 | 80600 | 80800 |
| 375.22 | 80600 | 80676 | 80600 | 80800 |
| 387.4 | 80657 | 80700 | 80600 | 80800 |
| 400 | 80694 | 80694 | 80683 | 80793 |

Supplementary D


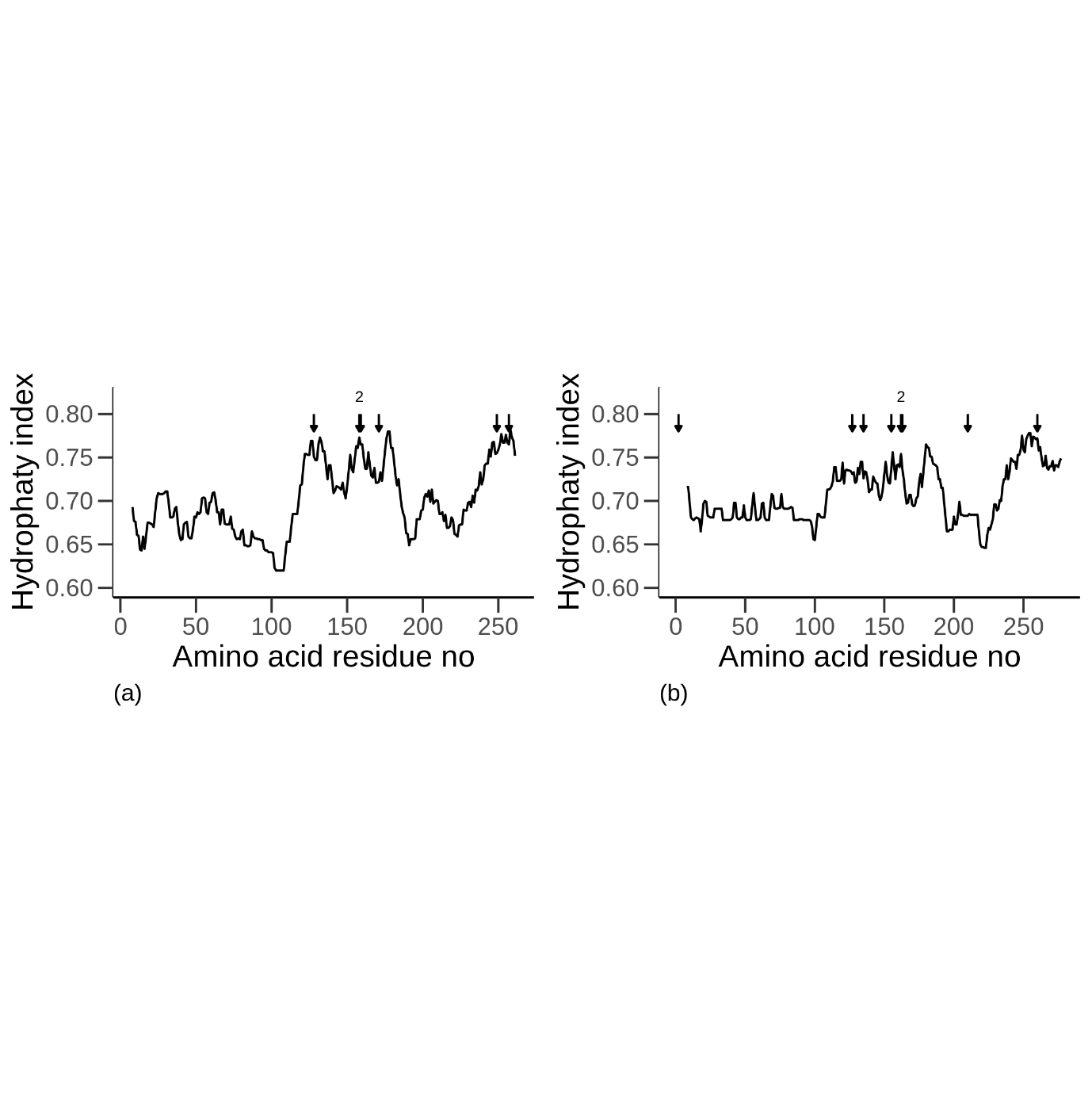


**Figure 11**. Hydropathy index (hydrophobicity for each amino acid with a window size of 15 amino acids) for each amino acid residue of (**a**) α-gliadin and (**b**) low molecular weight glutenin, indicating hydrophobic (+) and hydrophilic (-) regions in the protein. Cysteine amino acids are marked with an arrow, and the number 2 indicates two vicinal cysteines marked with two arrows at that position.

Figure 11 depicts the hydropathy index for the two proteins α-gliadin and LMW-GS, according to Rose et al. [56].

Supplementary E


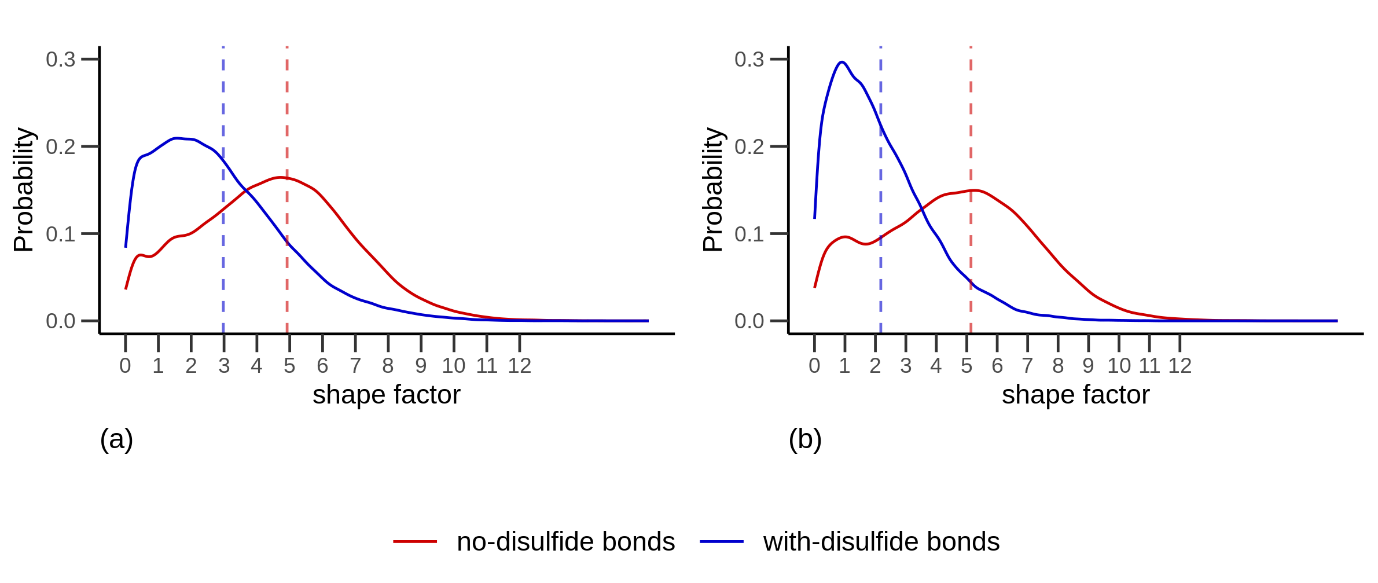


**Figure 12**. The shape factor of (**a)** α-gliadin and (**b**) LMW-GS, with disulphide bonds (red) and without (blue). Dotted lines highlight the mean values.

The shape factor distribution for α-gliadin and LMW-GS (figure 12), where low numbers indicate an average globular shape and high numbers indicate a rod-like shape.
