# Supplementary figures and images for: Glutenin and Gliadin, a Piece in the Puzzle of their Structural Properties in the Cell Described through Monte Carlo Simulations
Source: Biomolecules. 2020 Jul 23;10(8):1095. doi: 10.3390/biom10081095 (PMC7465137; doi:10.3390/biom10081095)

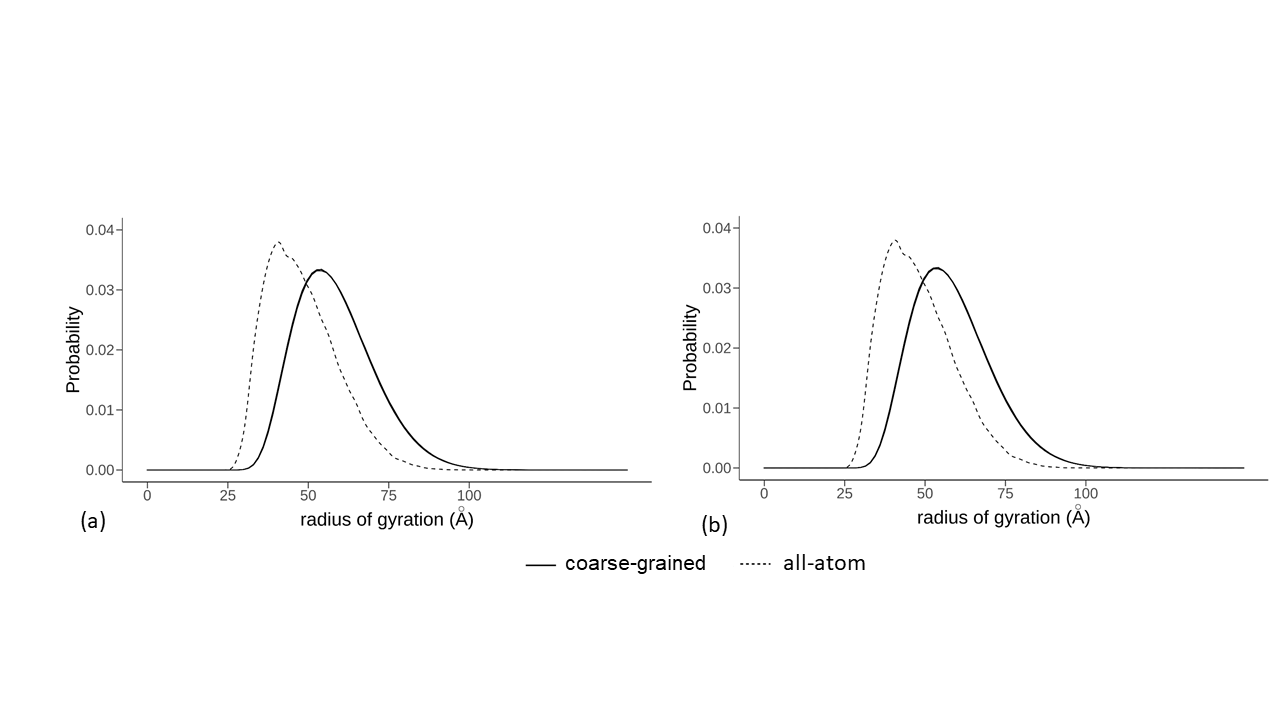

Supplement: Supplementary file 1 [file biomolecules-10-01095-s001.zip › supplementary/Figure10.tiff]

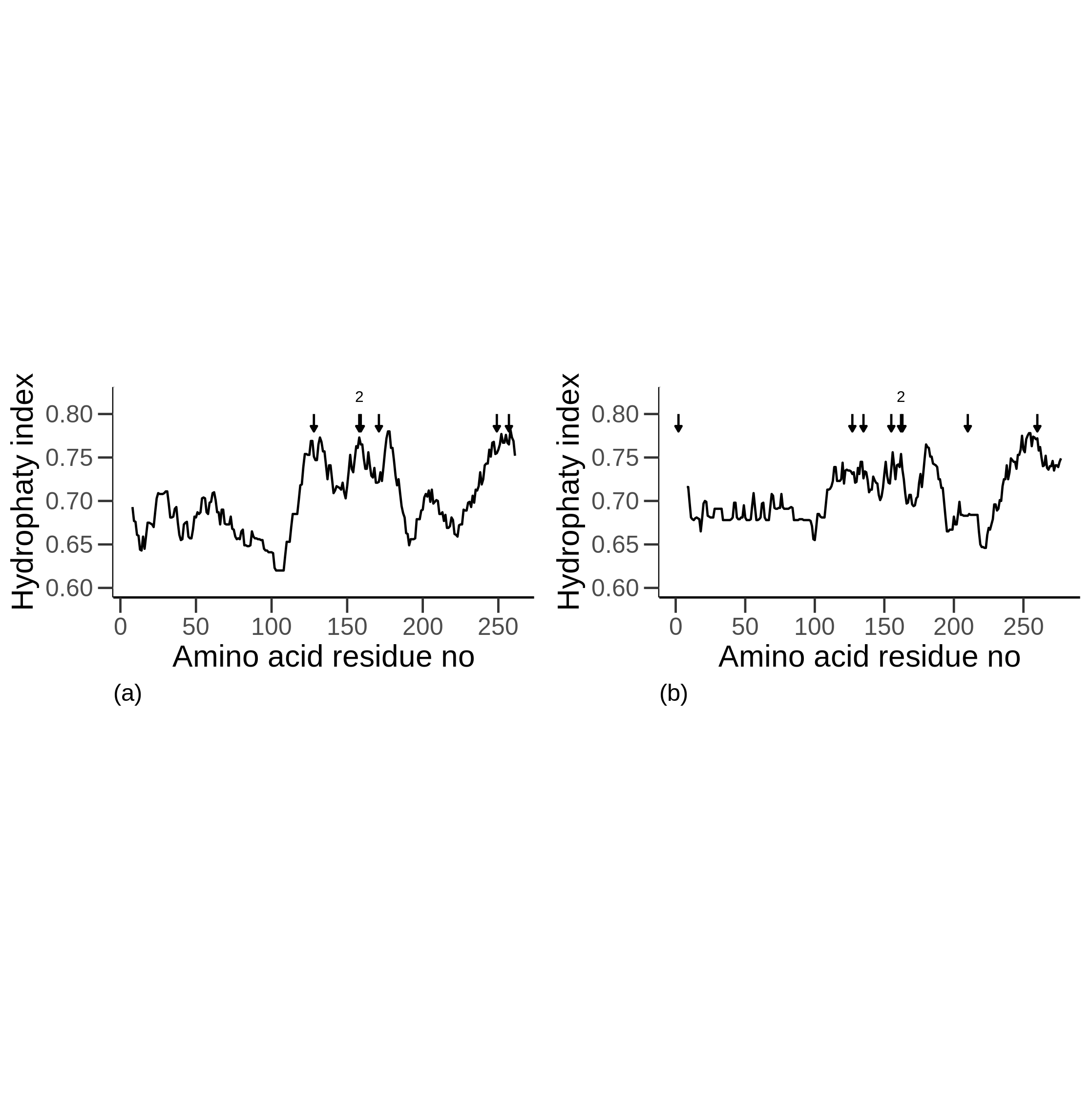

Supplement: Supplementary file 1 [file biomolecules-10-01095-s001.zip › supplementary/Figure11.tiff]

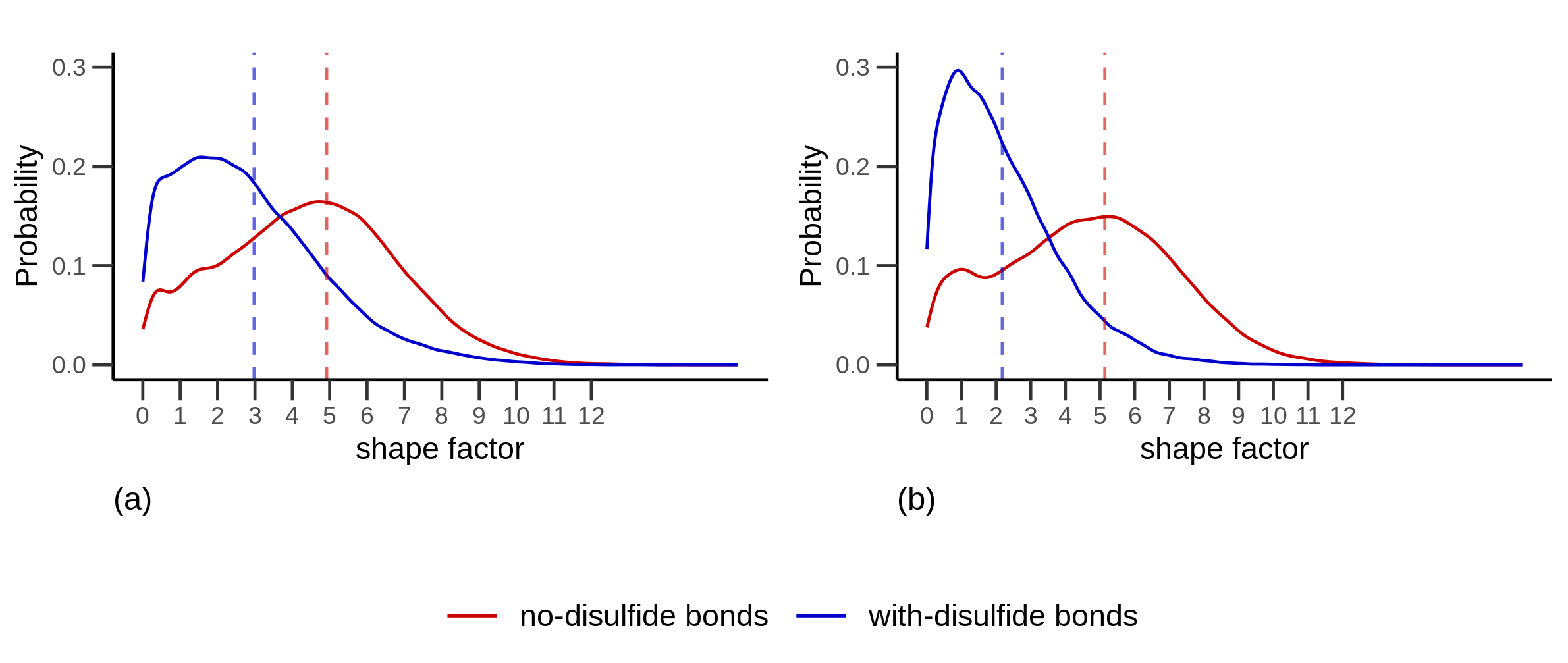

Supplement: Supplementary file 1 [file biomolecules-10-01095-s001.zip › supplementary/Figure12.tiff]
